# Pharmacogenetics aspects of oral anticoagulants therapy

**Published:** 2015

**Authors:** FC Militaru, SC Vesa, TR Pop, AD Buzoianu

**Affiliations:** * Department of Pharmacology, Toxicology and Clinical Pharmacology, “Iuliu Haţieganu” University of Medicine and Pharmacy Cluj-Napoca; ** 5th Department of Surgery, Municipal Hospital of Cluj-Napoca, “Iuliu Haţieganu” University of Medicine and Pharmacy Cluj-Napoca

**Keywords:** Vitamin K antagonists, Vitamin K antagonists, CYP2C9 gene, VKORC1 gene

## Abstract

**Rationale:** Vitamin K antagonists (VKA), such as warfarin and acenocoumarol, are widely used for the prevention and treatment of thromboembolic diseases and they are some of the most commonly prescribed types of medications. They are characterized by narrow therapeutic indices and inter-individual or intra-individual variability in response to the treatment.

**Objective:** to establish the influence of several genetic factors on VKA efficacy and adverse reactions.

**Methods and Results:** The metabolism of VKA differs depending on their chemical structure: indandiones derivatives (fluindione) or coumarin derivatives (acenocoumarol, phenprocoumon or warfarin). They are mostly metabolized in hepatocytes via a monooxygenase, cytochrome P450 2C9 (CYP2C9), resulting in inactive products. The gene encoding CYP2C9 is polymorphic, its genetic variants being associated with differences in the enzymatic activity of CYP2C9. The most important in terms of their frequency in the general population are CYP2C9*2 and CYP2C9*3. Both alleles are associated with a marked decrease in CYP2C9 enzyme activity. VK epoxide reductase (VKOR) is an enzyme with an important role in VK metabolism. Various polymorphisms in the VKORC1 gene have been described. VKORC1*2 haplotype seems to be the most important in relation to the variability in response to VKA.

**Discussions:** Various studies have shown a relationship between the genotype and the mean warfarin maintenance dosing: in patients carrying 2C9*1/ *2 alleles, the dose is reduced by 18-40% in patients carrying 2C9*2/ *2 alleles, by 21-49% in patients carrying 2C9*1/ *3 alleles. The A allele of the c.-1639G>A polymorphism in the VKORC1 gene is associated with the need for a lower dose of acenocoumarol in patients on anticoagulant therapy.

**Abbreviations:** SNP = Single Nucleotide Polymorphism, VKA = vitamin K antagonists, C1 - VKORC1 = vitamin K epoxide reductase complex subunit, INR = International Normalized Ratio

## Introduction

Patients with thromboembolic diseases such as pulmonary embolism, deep vein thrombosis or those with atrial fibrillation at risk of thromboembolic complications need short-term or sometimes long-term anticoagulation therapy.

Vitamin K antagonists (VKA), such as warfarin and acenocoumarol, are widely used for the prevention and treatment of thromboembolic diseases and they are some of the most commonly prescribed types of medications. They are characterized by narrow therapeutic indices and inter-individual or intra-individual variability in response to treatment [**1**,**2**].

Progress in pharmacogenetics may influence the improvement in the clinical approach to patients treated with VKA [**3**-**5**].

Pharmacogenetics and pharmacogenomics aim to establish the influence of genetic factors on drug efficacy and adverse reactions.

Genetic polymorphisms, SNPs (Single Nucleotide Polymorphism) being the most common, are minimal changes in genetic information, present in more than 1% of the population, considered to be normal variants, but nevertheless, in certain circumstances, they may have a phenotypic impact. These genetic polymorphisms make an important contribution to the great inter-individual and inter-ethnic variability in drug response [**6**].

**Metabolism of VKA**

The metabolism of oral anticoagulants differs depending on their chemical structure: indandiones derivatives (fluindione) or coumarin derivatives (acenocoumarol, phenprocoumon or warfarin). The most commonly used oral anticoagulants are coumarin derivatives. They are mostly metabolized in hepatocytes via a monooxygenase, cytochrome P450 2C9 (CYP2C9), resulting in inactive products [**7**]. CYP2C9 accounts for 20-25% of the hepatic cytochrome P450, being a protein that comprises 489 amino acids and whose gene is located on chromosome 10 [**8**]. CYP2C9 is involved in the metabolism of 15-20% of the drugs that are currently used in medical practice, some examples being: phenytoin, tolbutamide, glipizide, sartans, many of the non-steroidal anti-inflammatory drugs and oral anticoagulants [**9**]. It is known that the gene encoding CYP2C9 is polymorphic, its genetic variants being associated with differences in the enzymatic activity of CYP2C9. Studies carried out in different ethnic groups have revealed the existence of several allelic variants of the CYP2C9 gene, such as CYP2C9*2, CYP2C9*3, CYP2C9*4, CYP2C9*5, CYP2C9*6, and others. Of these, the most important in terms of their frequency in the general population are CYP2C9*2 and CYP2C9*3. Both alleles are associated with a marked decrease in CYP2C9 enzyme activity, with approximately 12% residual enzyme activity in the case of CYP2C9*2 and 5% in the case of CYP2C9*3 [**10**]. CYP2C9*2 and CYP2C9*3 variants are most common in Caucasians, with allelic frequencies of 10-14% (CYP2C9*2) and 8-10% (CYP2C9*3), compared to 1-2% (CYP2C9*2) and 0 (CYP2C9*3) in Asians, or 0.5-1% (CYP2C9*2) and 1% (CYP2C9*3) in Africans. In a study conducted in Romania, CYP2C9*2 allele was present in 11.3% of the subjects, while CYP2C9*3 allele was determined in 9.3% subjects [**11**]. Acenocoumarol is inactivated as a result of hydroxylation by CYP2C9, which is why individuals who carry at least one defective CYP2C9*2 allele, especially CYP2C9*3 (allele associated with only 5% CYP2C9 enzyme activity) are susceptible to excessive anticoagulation for average doses of acenocoumarol.

**VK epoxide reductase - pharmacological target of oral anticoagulants**

Oral anticoagulants factors lead to the synthesis of VK-dependent clotting factors (II, VII, IX and X), gamma-glutamyl carboxylase, functionally inactive. Physiologically, gamma carboxylation of glutamic acid residues of these factors is an essential post-translational maturation phase, allowing the binding of clotting factors to platelet phospholipids, via calcium ions. Gamma-carboxylation is done by an enzyme - gamma-glutamyl carboxylase, whose cofactor is the reduced form of VK. This is converted to VK epoxide, which requires VK recycling to its reduced form, VK quinone (K) and then VK hydroquinone (KH2), under the action of certain reductases that have not been clearly identified until recently [**12**]. In February 2004, in the same issue of *Nature* review, two research teams published the identification of the gene encoding VK epoxide reductase complex subunit C1 (VKORC1) [**13**,**14**]. The VKORC1 gene is located on chromosome 16 and encodes a dithiol-dependent reductase that converts VK epoxide to VK quinone. This enzyme appears to be one of the target enzymes of oral anticoagulants. Irreversible inhibition of VKORC1 by oral anticoagulants blocks VK regeneration, resulting in non-functional pro-coagulation factors.

Various polymorphisms in the VKORC1 gene have been described, most of them being grouped into 4 major haplotypes. Among them, VKORC1*2 haplotype seems to be the most important in relation to the variability in response to oral anticoagulants and the risk of excessive bleeding [**15**]. The VKORC1*2 haplotype is labelled by the c.G-1639A polymorphism located in the promoter region of the VKORC1 gene, indicating the presence of a low amount of active VK by disrupting its recycling mechanism via epoxide reductase. Recent studies have shown that the VKORC1*2 haplotype is associated with the risk of excessive anticoagulation in acenocoumarol average dose and thus, with bleeding events. The C1173T polymorphism in intron 1 of the VKORC1 gene is as representative for the VKORC1*2 haplotype as the c.G-1639A polymorphisms, because they are in complete linkage disequilibrium with each other [**16**]. Regarding the C1173T polymorphism, there is an approximately 45% T-allele frequency in Caucasians, which means that almost half of the individuals belonging to this population would be susceptible to an increased sensitivity to acenocoumarol. In a study on a population from Romania, the c.G-1639A polymorphism recorded a G allele frequency of 57.8% and an A allele frequency of 42.2% [**17**]. It seems that the VKORC1*2 haplotype has a greater contribution (40%) to the inter-individual and inter-ethnic variability in response to acenocoumarol than the CYP2C9 variants. Taking this into account, as well as the up to 14% contribution of the CYP2C9 variants, it appears that the variability in response to acenocoumarol is over 50% determined by CYP2C9 and VKORC1 variants [**18**]. If the VKORC1*2 haplotype is associated with the risk of excessive anticoagulation in case of average doses of oral anticoagulants, there are also rare mutations in the VKORC1 gene associated with anticoagulant resistance and with the need for higher doses of anticoagulant. Such a mutation is a g. G5417T transversion, which results in the substitution of an aspartate with a tyrosine at position 36 (p.Asp36Tyr) of the VKORC1 molecule, whose presence requires high doses of warfarin in order to trigger the anticoagulant effect [**19**]. It should be noted that the relationship between this mutation and the response to acenocoumarol is not known.

**Individual variability in response to the treatment with oral anticoagulants: environmental factors and VKORC1 and CYP2C9 gene polymorphisms**

The difficulty in managing oral anticoagulants is closely related to the narrow therapeutic index range of these drugs and to the great inter- and intra-individual variability in response to the treatment. This is estimated by measuring the International Normalized Ratio (INR), sensitive to clotting factors deficiencies (factors II, VII and IX, three of the VK-dependent clotting factors) [**7**].

For a long time, environmental factors were considered responsible for the inter- and intra-individual variations in the response to oral anticoagulant therapy. These factors include: patient characteristics (age, gender, body mass index], dietary intake of VK, comorbidities (liver failure, severe renal failure, heart failure, thyroid disease, etc.), acute inter-current pathologies (fever, sepsis, decompensated heart failure, diarrhoea, etc.) and concomitant drug therapy [**20**,**21**].

Along with demographic and environmental factors, genetic polymorphisms have also been identified, explaining part of the variability in response to oral anticoagulant therapy [**22**].

CYP2C9 polymorphisms vary according to ethnicity (Caucasian, African or Asian). The most common variants in Caucasians are CYP2C9*2 (Arg 144-Cys) and CYP2C9*3 (Ile 359-Leu), present in 8-19%, respectively 6-10% of the subjects, which indicates that almost a quarter of the general population have at least one mutant allele. The mutant enzymes resulting from these polymorphisms are less active than normal enzymes, resulting in a reduction in the metabolism of coumarin derivatives: subjects bearing at least one mutant allele have an increased sensitivity to these compounds and are referred to as “poor metabolizers” [**8**].

Various studies have shown a relationship between the genotype and the mean warfarin maintenance dosing [**23**-**27**]: in middle-aged patients, compared to patients carrying two normal alleles (2C9*1/*1) the dose is reduced by 13-22%; in patients carrying 2C9*1/*2 alleles, the dose is reduced by 18-40% in patients carrying 2C9*2/*2 alleles, by 21-49% in patients carrying 2C9*1/*3 alleles, by 18-73% in patients carrying 2C9*2/*3, and by 71% or more in patients carrying 2C9*3/*3 alleles. The results are comparable to those obtained in studies conducted on acenocoumarol and phenprocoumon [**28**-**30**]. Moreover, some studies have shown that the prevalence of CYP2C9*1/*3, CYP2C9*2/*2 and CYP2C9*2/*3 genotypes was higher in the group of patients treated with low doses of acenocoumarol compared to those treated with higher and medium doses [**17**,**31**].

In a retrospective study conducted on 185 patients receiving long-term warfarin therapy, with a mean follow-up of 13 months, Higashi et al. revealed that the possession of at least one CYP2C9 mutant allele is associated with a significantly increased risk of overdose (*hazard ratio* [HR] 1.40 [95% CI: 1.03-1.90]), with an increase in the maintenance dose range (HR 0.65 [95% CI: 0.45-0.94]) and especially with an increased risk of major bleeding (HR 2.39 [95% CI: 1.18-4.86]) [**22**]. Regarding the risk of overdose or the risk of bleeding, the results are comparable to those obtained in studies on the doses of acenocoumarol and phenprocoumon [**28**,**32**,**33**].

The discovery of polymorphisms in the VKORC1 gene is a step forward in understanding the inter-individual variability in oral anticoagulant maintenance dose range. In a study conducted on 147 patients treated with warfarin, D'Andrea et al. have identified a first polymorphism (1173C> T) as an independent factor influencing the mean daily maintenance dose: it is significantly lower in 1173TT patients (3.5 mg; p<0.001) than in 1173CT patients (4.8 mg; p=0.002) and 1173CC patients (6.2 mg) [**34**]. Militaru et al. have demonstrated the influence of the c.-1639G>A polymorphism on the time to therapeutic INR [**35**].

Rieder et al. have studied the VKORC1 gene thoroughly and identified two A and B haplotypes in 186 Caucasian subjects (including the polymorphism identified by D'Andrea et al.): A/ A subjects required a significantly lower warfarin maintenance dose (2.7 mg) than A/ B subjects (4.9 mg) and B/ B subjects (6.2 mg) [**36**].

Montes et al. have also shown that the A allele of the c.-1639G>A polymorphism in the VKORC1 gene is associated with the need for a lower dose of acenocoumarol in patients on anticoagulant therapy [**37**].

Due to the difficult management of VKA and the risk of bleeding and thromboembolic events, there have been attempts to develop algorithms in order to establish the therapeutic dose that would protect the patient from these risks. The algorithms developed to estimate the stable therapeutic doses of warfarin, acenocoumarol or phenprocoumon are based both on clinical and pharmacological characteristics of the patients (age, gender, body mass index, concomitant therapy with amiodarone, statins, antifungals, antibiotics, ACE inhibitors, liver failure, kidney failure) and on mutations that directly or indirectly influence the therapy with VKA (polymorphisms in the VKORC1, CYP2C9, CYP4F2, and GGCX genes) [**38**-**43**]. Although the algorithms establishing the stable dose of VK antagonists have shown good results in reducing the frequency of adverse reactions, studies did not indicate a good cost-efficiency ratio.

## Conclusion

The management of VKA is complex and it depends of several mutations for CYP2C9 and VKORC1 genes.

**Acknowledgment**

This paper was published under the frame of European Social Fund, Human Resources Development Operational Programme 2007-2013, project no. POSDRU/159/1.5/S/138776
